# Intermittent Hypoxia-Induced Cardiomyocyte Death Is Mediated by HIF-1 Dependent MAM Disruption

**DOI:** 10.3390/antiox11081462

**Published:** 2022-07-27

**Authors:** Sophie Moulin, Amandine Thomas, Stefan Wagner, Michael Arzt, Hervé Dubouchaud, Frédéric Lamarche, Sophie Bouyon, Guillaume Vial, Diane Godin-Ribuot, Jean-Louis Pépin, Claire Arnaud, Elise Belaidi

**Affiliations:** 1 Univ. Grenoble Alpes, HP2, F-38042 Grenoble, France; moulin.sophie07@laposte.net (S.M.); amandine.thomas-zanetti@univ-lyon1.fr (A.T.); sophie.bouyon@univ-grenoble-alpes.fr (S.B.); guillaume.vial@univ-grenoble-alpes.fr (G.V.); diane.ribuot@univ-grenoble-alpes.fr (D.G.-R.); jpepin@chu-grenoble.fr (J.-L.P.); claire.arnaud@univ-grenoble-alpes.fr (C.A.); 2 INSERM, U1300, F-38042 Grenoble, France; 3 Department of Internal Medicine II, University Hospital Regensburg, 93053 Regensburg, Germany; stefan.wagner@ukr.de (S.W.); michael.arzt@klinik.uni-regensburg.de (M.A.); 4 Univ. Grenoble Alpes, LBFA, INSERM, U1055, F-38958 Grenoble, France; herve.dubouchaud@univ-grenoble-alpes.fr (H.D.); frederic.lamarche@univ-grenoble-alpes.fr (F.L.); 5 Centre Hospitalier Universitaire des Alpes, F-38042 Grenoble, France

**Keywords:** sleep disordered breathing, intermittent hypoxia, hypoxia inducible factor-1, mitochondria associated-ER membrane, cardiomyocyte death

## Abstract

Rationale: Intermittent hypoxia (IH) is one of the main features of sleep-disordered breathing (SDB). Recent findings indicate that hypoxia inducible factor-1 (HIF-1) promotes cardiomyocytes apoptosis during chronic IH, but the mechanisms involved remain to be elucidated. Here, we hypothesize that IH-induced ER stress is associated with mitochondria-associated ER membrane (MAM) alteration and mitochondrial dysfunction, through HIF-1 activation. Methods: Right atrial appendage biopsies from patients with and without SDB were used to determine HIF-1α, Grp78 and CHOP expressions. Wild-type and HIF-1α^+/−^ mice were exposed to normoxia (N) or IH (21–5% O_2_, 60 cycles/h, 8 h/day) for 21 days. Expressions of HIF-1α, Grp78 and CHOP, and apoptosis, were measured by Western blot and immunochemistry. In isolated cardiomyocytes, we examined structural integrity of MAM by proximity ligation assay and their function by measuring ER-to-mitochondria Ca^2+^ transfer by confocal microscopy. Finally, we measured mitochondrial respiration using oxygraphy and calcium retention capacity (CRC) by spectrofluorometry. MAM structure was also investigated in H9C2 cells incubated with 1 mM CoCl_2_, a potent HIF-1α inducer. Results: In human atrial biopsies and mice, IH induced HIF-1 activation, ER stress and apoptosis. IH disrupted MAM, altered Ca^2+^ homeostasis, mitochondrial respiration and CRC. Importantly, IH had no effect in HIF-1α^+/−^ mice. Similar to what observed under IH, HIF-1α overexpression was associated with MAM alteration in H9C2. Conclusion: IH-induced ER stress, MAM alterations and mitochondrial dysfunction were mediated by HIF-1; all these intermediate mechanisms ultimately inducing cardiomyocyte apoptosis. This suggests that HIF-1 modulation might limit the deleterious cardiac effects of SDB.

## 1. Introduction

Sleep disordered breathing (SDB) is one of the most frequent chronic pathologies, affecting one billion people in the world [1]. Chronic intermittent hypoxia (IH), the key feature of SDB, is described as the major detrimental factor responsible for the cardiovascular and metabolic consequences. Whereas it is difficult to clearly demonstrate that patients with longstanding SDB develop heart failure, it is now well described that OSA patients exhibit cardiac remodeling (i.e., left ventricular hypertrophy and dilation) and subclinical markers of diastolic dysfunction, which are correlated to the severity of IH [2,3]. In addition, IH also induces a reduction in myocardial salvage and an increase in infarct size following myocardial infarction (MI) [4,5]. Accordingly, in pre-clinical models, rodents exposed to chronic IH exhibit a decrease in cardiomyocyte viability [6,7] and an increase in infarct size after myocardial ischemia–reperfusion (I/R) [8,9,10,11,12]. Unfortunately, recent randomized controlled trials failed to demonstrate a significant reduction in cardiovascular events by the gold standard continuous positive airway pressure (CPAP) treatment [13,14]. Therefore, a better knowledge of underlying mechanisms linking chronic IH and cardiomyocyte death is crucial to propose alternative or combined treatment with CPAP.

One of the main actors of IH-induced cardiomyocyte death is the hypoxia inducible factor-1 (HIF-1). HIF-1 is a transcription factor composed of two sub-units, HIF-1β and HIF-1α, the O_2_-sensitive sub-unit. Briefly, in normoxia, HIF-1α is hydroxylated by prolyl-4-hydroxylases (PHD), recognized by the Von Hippel Lindau peptide, ubiquitinylated and then degraded by the proteasome. Under hypoxia, PHD are inhibited, leading to HIF-1α nuclear translocation and to its association with HIF-1β inducing subsequent HIF-1 activation [15]. Once activated, HIF-1 induces both adaptive and maladaptive responses. HIF-1 plays an important role in cardioprotective strategies such as ischemic [16], hypoxic [17] or pharmacological [18] preconditioning, where it is required to decrease infarct size following ischemia–reperfusion. Conversely, chronic HIF-1 activation is recognized to induce deleterious effects. Indeed, sustained HIF-1α overexpression leads to cardiac hypertrophy [19], and we demonstrated that chronic IH is a potent inducer of its adverse effects on infarct size [20,21]. In line, we and others demonstrated an important cooperation between HIF-1 and ER stress in these detrimental effects. In the brain, inhibition of CHOP expression prevents IH-induced HIF-1α expression, oxidative stress and neural injury [22]. In the lung, Debrel et al. demonstrated that IH-induced ER stress and HIF-1 transcriptional activation, which were reciprocally inhibited by both HIF-1α silencing or ER stress inhibition, results in decreased apoptosis [23,24]. In the heart, inhibiting ER stress with tauroursodeoxycolic acid (a chemical chaperone known to improve protein folding) prevents IH-induced HIF-1 activation and subsequent myocardial infarct size enhancement [25]. Moreover, under chronic IH, HIF-1 binds to the activating transcription factor 4 (ATF4) to induce pro-apoptotic expression of C/EBP homologous protein (CHOP) and cardiomyocyte death [9]. Thus, the reciprocal relationship between HIF-1 and ER stress is involved in IH-induced cell death, but the elucidation of mechanism involved in their inter-relation is still missing. Considering the well-described relationship between ER stress, calcium homeostasis and mitochondria homeostasis [26], we hypothesized that mitochondria could represent a mechanistic node that could contribute to IH-induced myocardial cell death.

Mitochondrial homeostasis maintenance is essential for cardiomyocyte life or death [27]. In the heart, the abundant proportion of mitochondria could explain cardiomyocyte vulnerability to oxidative stress and calcium homeostasis alterations, and consequently to IH. The impact of IH on mitochondria has been poorly investigated. IH induces a decrease in mitochondrial membrane potential in pulmonary cells [28] and a reduction in O_2_ consumption, associated with an increase in ROS production in the cerebral cortex from neonatal rodents [29,30,31]. However, mitochondrial function has yet to be studied in cardiomyocytes in the context of IH.

Over the past two decades, the mitochondria-associated ER membrane (MAM) has been studied to understand how ER and mitochondria communicate and regulate calcium homeostasis and ensure ER and mitochondria homeostasis [32]. One of the most studied MAM complexes in the myocardium is composed of the mitochondrial voltage dependent anion channel (VDAC), the inositol phosphate 3 receptor (IP3R) and the bridge protein glucose regulated protein 75 (Grp75). Depending on the context (stress), changes in MAM integrity lead to opposite effects on cardiomyocytes. Paillard et al. demonstrated that acute MAM disruption (by blocking Grp75) was protective against myocardial ischemia-reperfusion injury [33]. Conversely, the same team recently demonstrated that reduction in ER-mitochondria Ca^2+^ transfer by MAM disruption induces early mitochondrial dysfunction and contributes to chronic disease, such as diabetic cardiomyopathy [34]. Therefore, fine regulation of this complex is mandatory to maintain functionality of both ER and mitochondria in order to prevent ER stress and/or mitochondria-dependent cell death [35]. Of note, HIF-1 is a key player of mitochondrial homeostasis maintenance and its activation also depends on energetic cell status. Indeed, when O_2_ partial pressure decreases, HIF-1 favors anaerobic glucose homeostasis, modifies the respiratory chain complex constitution, and inhibits the transformation of pyruvate in acetyl CoA in order to preserve mitochondria [15]. However, little is known about its role in chronic-IH induced potential mitochondrial alteration in the heart. In the present study, we propose to explore if IH-induced ER stress is associated with MAM integrity alteration and mitochondrial dysfunction in the heart, through HIF-1 activation. Using human atrial samples, wild-type and HIF-1α heterozygous mice, as well as H9C2 cells, we aimed to investigate the mechanistic HIF-1-dependant cascade of cardiomyocyte death under chronic IH exposure through: (1) validation of HIF-1 activation in human and rodent myocardial tissue, (2) evaluation of pro-apoptotic ER stress, (3) determination of MAMs disruption, and (4) consequences on mitochondrial function.

## 2. Materials and Methods

### 2.1. Atrial Biopsies from SDB Patients

Thirty-nine consecutive patients undergoing elective coronary artery bypass graft (CABG) were enrolled between October 2014 and March 2015 at the University Hospital Regensburg (Regensburg, Germany). Left auricle appendage biopsies were sampled. Assessment of SDB was performed using the ApneaLink device (ResMed Inc., Martinsried, Germany) [36,37]. The institutional ethics committee of the University of Göttingen, in agreement with the Declaration of Helsinki, approved the procedures (approval number No. 14/09/11). Patients were stratified in quartiles of AHI. According to our “moderate to severe” rodent model [38], the fourth quartile with a moderate to severe degree of SDB (AHI 22.2 ± 0.6/h, SDB) was compared with the first quartile without SDB (AHI 4.6 ± 0.6/h, Control) (Figure S1).

### 2.2. Rodent Model of Intermittent Hypoxia

#### 2.2.1. Animals

The protocol was approved by the French Minister (number: 2015061813053181). All experiments were performed in accordance with the European Convention for the Protection of Vertebrate Animals Used for Experimental and Other Scientific Purposes (decree 2013-118 and orders of 1 February 2013, in accordance with the NIH *Guide for the Care and Use of Laboratory Animals*). Male Swiss/SV129 mice with a heterozygous deletion of the gene encoding for the O_2_-sensitive HIF-1α subunit of HIF-1 (HIF-1α^+/−^) and their wild-type (HIF-1α^+/+^) were furnished by “Plateforme de Haute Technologie Animale”, UGA.

#### 2.2.2. Experimental Design

**Figure 1:**
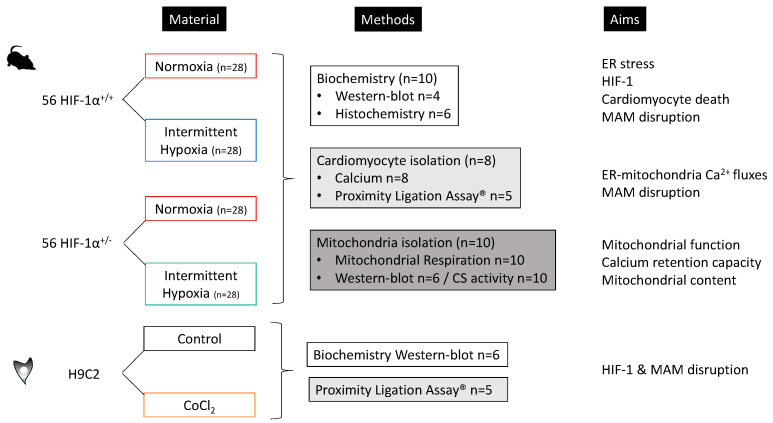
Experimental design: repartition of wild-type mice SwissxSV129 (HIF-1α^+/+^) and HIF-1α heterozygous mice (HIF-1α^+/−^) in normoxia and intermittent hypoxia during 21 days before heart harvesting, in order to explore the impact of IH on endoplasmic reticulum (ER), ER-mitochondria Ca^2+^ fluxes, mitochondria and mitochondria-associated ER membrane (MAM) homeostasis (biochemical analysis, cardiomyocyte isolation and mitochondria isolation); in vitro model using H9C2 cardiomyoblast treated, or not, with CoCl_2_ (100 uM, 1 h), in order to validate the impact of HIF-1 activation on MAM integrity (Western blot and proximity ligation assay).

One week after arrival, mice were randomly assigned to be exposed to normoxia (N) or intermittent hypoxia (IH). One hundred and twelve mice were separated in 4 groups (n = 28 per group): HIF-1α^+/+^ -N, HIF-1α^+/+^ -IH, HIF-1α^+/−^ -N and HIF-1α^+/−^ -IH. Three experimental sets were designed (Figure 1). The 1st set was used for biochemistry; the 2nd set was used to isolate cardiomyocytes in order to assess MAM integrity and Ca^2+^ fluxes between ER and mitochondria; the 3rd set was used for mitochondrial assessments.

#### 2.2.3. Chronic Intermittent Hypoxia

Animals were exposed to 21 d of N or IH (8 h/d-6 a.m./2 p.m.). The IH stimulus consisted of 60 s cycles (30 s of 5% FiO_2_ and 30 s of 21% FiO_2_), as previously described [10].

#### 2.2.4. In Vitro Model, Cell Culture and Design

H9C2 cardiomyoblasts were cultivated in classic conditions (DMEM high glucose, 10% SVF, 1% penicillin/streptomycin, 37 °C, 5% CO_2_) in presence or absence of CoCl_2_ (1 mM, 1 h, n = 5), used as a potent HIF-1 activator, in order to determine HIF-1α and Grp75 expression (n = 6), as well as MAM integrity (n = 5), Figure 1.

#### 2.2.5. HIF-1α, ER Stress Markers and Apoptosis

Western blot analyses of HIF-1α, CHOP, Grp78, and Grp75 were performed on whole protein extracted from hearts of N and IH mice as previously described [10]. Immunochemistry analyses of HIF-1α, Grp78 and CHOP were performed on heart cryosections of HIF-1α^+/−^ and HIF-1α^+/+^ as previously described [10]. Cryosections were also used to detect apoptosis using terminal deoxynucleotidyl transferase-mediated dUTP nick end-labeling assay (Abcam, Cambridge, UK), following the manufacturer’s instruction (for more information, see Supplemental Material).

### 2.3. Ca^2+^ Inter-Organelle Fluxes and Assessment of MAM Integrity

#### 2.3.1. Cardiomyocyte Isolation

Hearts were retrogradely perfused in Langendorff mode, and cardiomyocytes were isolated using research grade Liberase™ (Roche Diagnostics, Meylan, France) as previously described [25,34]. Then, cells were seeded on Lab-Tek^®^ to process to inter-organelles calcium fluxes measurement.

#### 2.3.2. Confocal Microcopy for Inter-Organelles Ca^2+^ Fluxes

Cardiomyocytes were loaded with both Fluo4-AM/Rhod2-AM, or Fluo5-AM/Rhod2-AM as previously described [25,39]. Fluo4-AM, Fluo5-AM and Rhod2-AM are fluorescence-based indicators of cytosolic, ER and mitochondrial calcium variation, respectively. After baseline fluorescence recording, histamine was added to induce calcium release from the ER. Fluorescence slope was calculated between 250 s and 50 s after histamine application.

#### 2.3.3. Proximity Ligation Assay and Immunoprecipitation for MAM Integrity

Duolink^®^ Proximity Ligation Assay (St Quentin-Fallavier, France) was performed on isolated and seeded cardiomyocytes to detect proximity (<4 nm) between VDAC and IP3R1, following the manufacturer’s instructions. Immunobinding of VDAC was performed on IP3R1-immunoprecipitated proteins.

### 2.4. Mitochondrial Exploration

#### 2.4.1. Oxidative Phosphorylation

Mitochondria were isolated using differential centrifugation as previously described [40]. NADH-linked and FADH_2_-linked mitochondrial respiration were assessed by measuring O_2_ consumption by oxygraphy with a Clark electrode. Mitochondrial content was assessed by performing Western blot of respiratory chain complexes and citrate synthase activity measurement as previously described [41].

#### 2.4.2. Calcium Retention Capacity

Calcium retention capacity (CRC) was also measured on isolated mitochondria using the calcium green 5-N, as previously described [40]. Briefly, Ca^2+^ pulses were added every minute to isolated mitochondria in a tank for spectrophotometry. Following enough calcium loading (corresponding to CRC), a massive release of the accumulated Ca^2+^ was detected by a brutal increase in extra-mitochondrial Ca^2+^ concentration indicating mitochondrial permeability transition pore (mPTP) opening.

### 2.5. Statistical Analysis

All analyses were performed with GraphPad prism 6.0 Software. For human samples, t-test was performed. For mice samples, two-way ANOVA followed by Tukey’s post-hoc was performed when normality was accepted (Shapiro–Wilkinson test). In other conditions, Kruskal–Wallis one-way ANOVA was followed by Dunn’s post-hoc test. Post-hoc tests were performed after a statistical significance of two-way ANOVA and Kruskal–Wallis one-way ANOVA for *p* value < 0.05. For post-hoc tests, statistical significance was set for *p* values < 0.05.

Material and Methods details are added in the online Supplemental Material section.

## 3. Results

### 3.1. Sleep Apnea in Patients and Intermittent Hypoxia in Rodents Increase HIF-1α Expression and Induces a Pro-Apoptotic ER Stress

**Figure 2:**
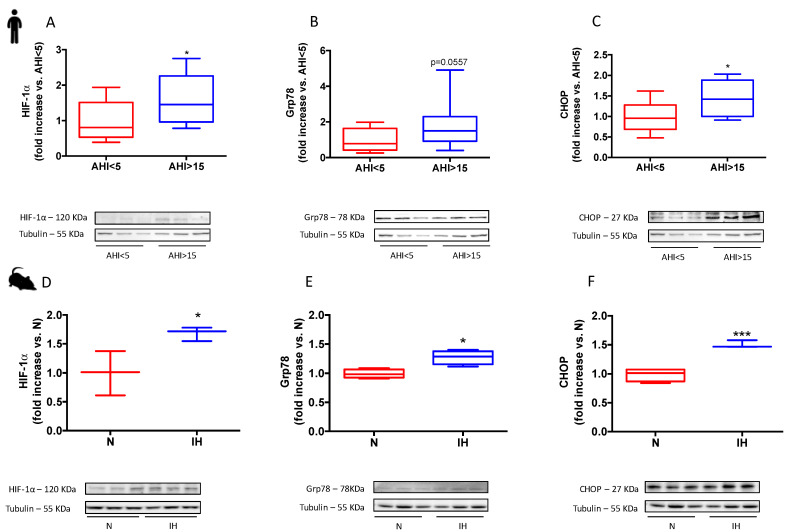
HIF-1 and ER stress in humans and mice under IH. Hypoxia inducible factor-1α (HIF-1α), glucose regulated protein-78 KDalton (Grp78) and C/EBP homologous protein (CHOP, copyright from Moulin S. et al., Can. J. Cardiol, 2020) expression relative to Tubulin. Patients were stratified in quartiles of AHI (n = 10 each), and lowest and highest quartiles were compared. Quantification and representative images for HIF-1α (**A**), Grp78 (**B**), and CHOP (**C**), in patients, respectively; quantification and representative images for HIF-1α (**D**), Grp78 (**E**), and CHOP (**F**), in mice, respectively. Data are expressed in median and min to max values and analyzed using t-test; * *p* < 0.05, *** *p* < 0.001.

Expressions of HIF-1α, Grp78 (the main ER stress sensor) and CHOP (the terminal pro-apoptotic factor of the unfolded protein response), in human atrial appendage biopsies of patients with sleep apnea (AHI > 22 ± 2 events.h^−1^) and in hearts from rodents exposed to chronic intermittent hypoxia are presented in Figure 2. HIF-1α, Grp78 and CHOP expressions were increased in atrial biopsies from apneic patients (Figure 2A–C) as well as in heart from mice submitted to IH (Figure 2D–F).

### 3.2. Intermittent Hypoxia Induces ER Stress and Apoptosis through HIF-1 Activation

**Figure 3:**
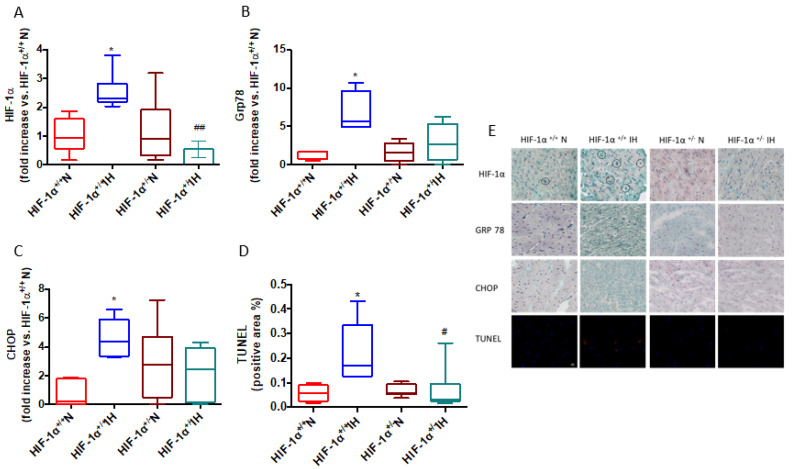
IH-induced ER stress and apoptosis is mediated by HIF-1. Hypoxia inducible factor-1α (**HIF-1α**, **A**), glucose regulated proteine-78 KDalton (**Grp78**, **B**), C/EBP homologous protein expression (**CHOP**, **C**), and terminal deoxynucleotidyl transferase dUTP nick end labeling (TUNEL) counterstained with DAPI (**D**), in HIF-1α^+/+^ and HIF-1α^+/−^ mice submitted to normoxia (N) or chronic intermittent hypoxia (IH), n = 4–6. Representative images (×20) for HIF-1α, Grp78, CHOP and TUNEL immunostainings in all groups (**E**). Data are expressed in median and min to max values and analyzed using Kruskal–Wallis followed by Dunn’s test; * *p* < 0.05 vs. IH; # *p* < 0.05 and ## *p* < 0.01 vs. HIF-1α^+/+^.

Figure 3 depicts the impact of partial HIF-1α gene deletion on ER stress and apoptosis in mice exposed to IH. IH increased nuclear HIF-1α expression (Figure 3A). Figure 3B,C show that IH failed to increase Grp78 and CHOP expressions in HIF-1α^+/−^ mice. Most importantly, apoptosis was significantly prevented in HIF-1α^+/−^ submitted to IH, compared with HIF-1α^+/+^ mice (Figure 3D). These results suggest that HIF-1, per se, plays a major role in IH-initiated ER stress and apoptosis.

### 3.3. HIF-1 Is Involved in IH-Induced MAM Disruption and Alteration of Calcium Homeostasis

**Figure 4:**
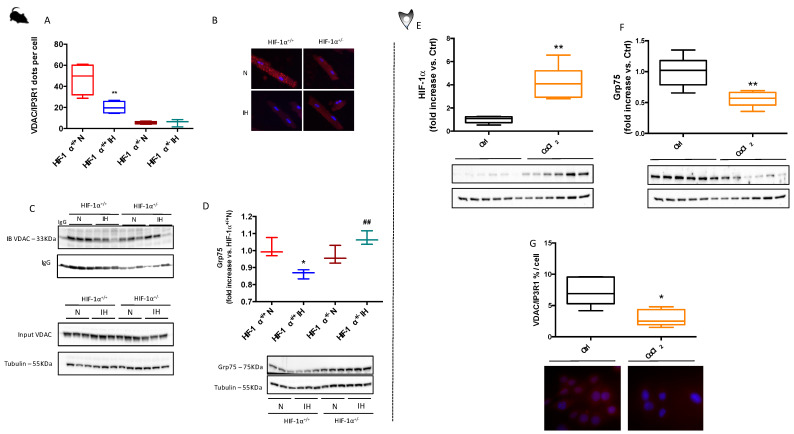
IH-induced MAM structural alteration is mediated by HIF-1. Left panels: HIF-1α^+/+^ and HIF-1α^+/−^ mice submitted to normoxia (N) or chronic intermittent hypoxia (IH). Quantification (**A**), and representative image (**B**), of voltage anion dependent channel (VDAC) and Inositol tri-Phosphate Receptor 1 (IP3R1) dots following proximity ligation assay (proximity < 0.4 nm in red; nucleus in blue-DAPI, n = 4–5, and immunoprecipitation of IP3R1 linked-protein followed by VDAC immunobinding (IB) (**C**), n = 3. Quantification and representative image of glucose regulated protein-75 KDalton (Grp75) (**D**), n = 3. Right panels: H9c2 cells stimulated by cobalt chloride (CoCl_2_, 1 mM, 1 h). Quantification and representative images of hypoxia inducible factor 1α (HI©α) (**E**), and Grp75 (**F**), expression, and VDAC/IP3R1 proximity (**G**), n = 5. Data are expressed in median and min to max values and analyzed using Kruskal–Wallis followed by Dunn’s test; * *p* < 0.05; ** *p* < 0.01 vs. N; ## *p* < 0.01 vs. HIF-1α^+/+^.

MAM disruption and consecutive alterations of calcium homeostasis are known to be involved in cardiomyocyte death. Here, under IH, proximity ligation assay and immunoprecipitation demonstrated a decrease in VDAC and IP3R1 proximity (Figure 4A,B) and interaction (Figure 4C). Interestingly, in HIF-1α^+/−^, mice, IH did not affect VDAC and IP3R1 interaction. Figure 3D demonstrates that IH also decreased Grp75 expression in HIF-1α^+/+^ mice, which was restored in HIF-1α^+/−^. Taken together, these results indicate that chronic IH leads to HIF-1-dependent-MAM disruption. In accordance, using H9c2 stimulated with CoCl_2_ that drastically increases HIF-1α expression (Figure 3E), we observed a decrease in Grp75 expression (Figure 4F), as well as a decrease in VDAC and IP3R1 proximity (Figure 4G).

Since MAM is functionally involved in mitochondrial Ca^2+^ homeostasis, we next investigated the Ca^2+^ transfer between ER and mitochondria in HIF-1α^+/+^ and HIF-1α^+/−^ mice.

**Figure 5:**
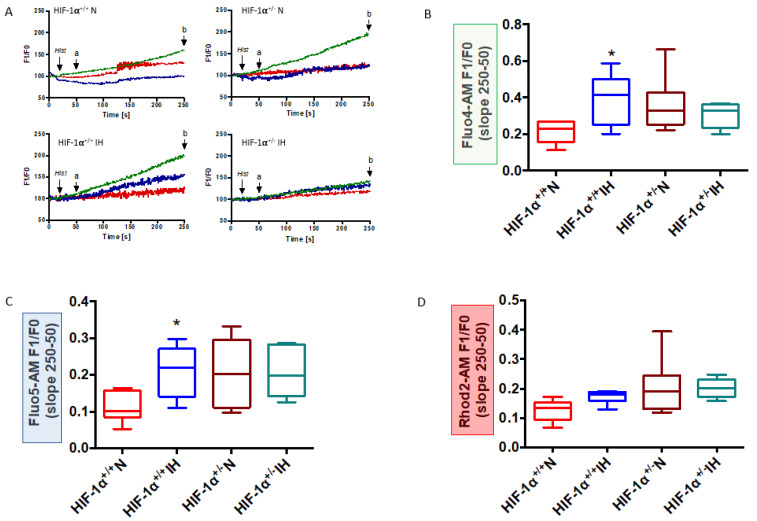
IH-induced Ca^2+^ homeostasis alteration is mediated by HIF-1. Mitochondrial-associated ER membrane calcium fluxes between ER and mitochondria in HIF-1α^+/+^ and HIF-1α^+/−^ mice submitted to normoxia (N) or chronic intermittent hypoxia (IH). Illustration of calcium fluorescence after histamine-induced ER-calcium leakage using Fluo 4-AM (cytosol, green), Fluo 5-AM (ER, blue) and Rhod 2 (mitochondria, red) recorded between 50 (a) and 250 (b) seconds after (**A**), n = 7–8. Quantification of fluorescence F1 relative to F0 recorded in cytosol (**B**), ER (**C**), and mitochondria (**D**). Data were expressed in median and min to max values and analyzed using Kruskal–Wallis followed by Dunn’s test; * *p* < 0.05 vs. N.

In HIF-1α^+/+^ N mice, histamine-induced Ca^2+^ leakage from the ER (blue line) resulted in cytosolic (green line) and mitochondrial (red line) Ca^2+^ loading (Figure 5A). The ability of histamine to deplete ER Ca^2+^ stores was decreased by IH (HIF-1α^+/+^ IH, Figure 5A, situation a). At the end of recording (Figure 5A, situation b), IH resulted in histamine-induced Ca^2+^ overload in cytosol (Figure 5B) and in ER (Figure 5C), but not in mitochondria (Figure 5C,D). There were no differences in cytosolic, ER and mitochondrial calcium loadings in HIF-1α^+/−^ N and HIF-1α^+/−^ IH mice after ER leakage (Figure 5B–D).

Altogether, these results suggest that HIF-1 plays a major role in IH-induced MAM disruption and mitochondrial Ca^2+^ homeostasis. Thereafter, we questioned its role in IH-induced mitochondrial dysfunction.

### 3.4. HIF-1 Is Involved in IH-Induced Mitochondrial Respiration Alteration

**Figure 6:**
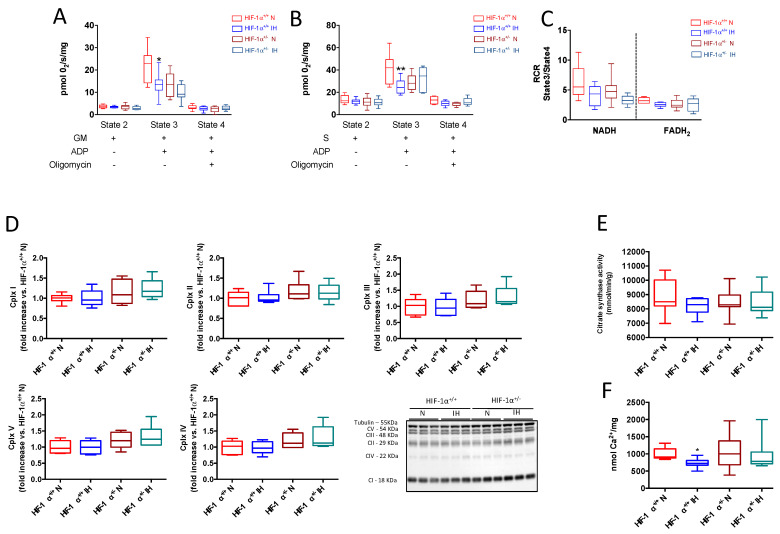
IH-induced mitochondrial respiration alteration is mediated by HIF-1. Mitochondrial oxygen (O2) consumption measured on isolated mitochondria from HIF-1α^+/+^ and HIF-1α^+/−^ mice submitted to chronic intermittent hypoxia (IH), following sequential addition of substrate (state 2), ADP (state 3, maximal respiration) and oligomycin (state 4). Quantification of O_2_ consumption for NADH-linked mitochondrial respiration (complex I-glutamate-malate GM) (**A**), and for FADH2-linked mitochondrial respiration (complex II-succinate in presence of complex I inhibition by rotenone) (**B**). Respiratory control ratio (RCR) calculated as the ratio of O2 consumption in state 3 relative to state 4 for NADH and FADH2-linked mitochondrial respiration (**C**), n = 7–10. Quantification of each respiratory chain complex and representative image (**D**), n = 6. Citrate synthase activity (**E**), n = 7–10. Calcium retention capacity expressed in nmol Ca^2+^ per mg of mitochondrial protein content, on isolated mitochondria in presence of cyclosporin A (CsA, 1μM) (**F**). Data are expressed in median and min to max values and analyzed using Kruskal–Wallis followed by Dunn’s test; * *p* < 0.05, ** *p* < 0.01 vs. N.

In HIF-1α^+/+^ mice, chronic IH exposure reduced O_2_ maximal consumption around 38%, compared with the normoxic condition when mitochondrial respiration was induced by addition of complex I substrates (glutamate and malate) and ADP (state 3) (Figure 6A). Chronic IH also reduced O_2_ maximal consumption around 40% when mitochondrial respiration was triggered by addition of complex II substrate (succinate) (Figure 6B). The respiratory control ratio (RCR, obtained by dividing state 3/state 4) reflects mitochondrial capacity regarding ATP turnover in response to metabolic demand. Here, we observed a trend toward a decrease in RCR in hypoxic mice compared with normoxic mice (−35% and −22% for complex I and II, respectively, Figure 6C). These results mainly suggest that NADH and FADH_2_-linked maximal respiration were impaired by IH, whereas neither respiratory chain complex content nor citrate synthase activity demonstrated any modifications of mitochondrial content between groups (Figure 6D,E, respectively). In HIF-1α^+/−^ mice, maximal O_2_ consumption was similar in N and IH groups (Figure 6A,B). Taken together, these results indicate that HIF-1 is involved in IH-induced mitochondrial respiration alteration in the heart. One of the most important parameters linking mitochondrial respiration, Ca^2+^ homeostasis alteration and cardiomyocytes death, is the ability of mitochondria to maintain its susceptibility to Ca^2+^ loading. As expected, Figure 6F demonstrates that, under IH, mPTP from IH mice opens earlier than mPTP from N mice, which is no more the case in HIF-1α^+/−^ mice.

## 4. Discussion

**Figure 7:**
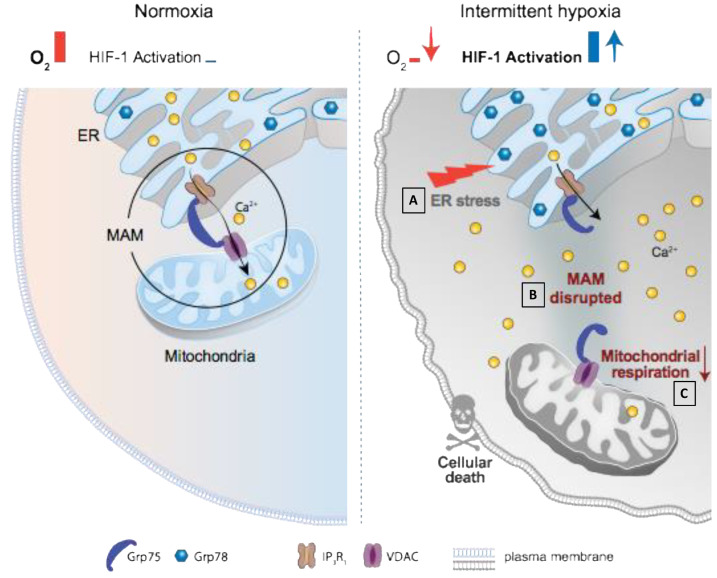
Graphical abstract. Normoxia is represented on the left panel. The communication between endoplasmic reticulum (ER) and mitochondria through the mitochondria-associated ER membrane (voltage anion dependent channel (VDAC), Inositol tri-Phosphate Receptor 1 (IP3R1), and glucose regulated protein-75 KDalton (Grp75)) maintains Ca^2+^ homeostasis, ER homeostasis and cell life. On the right panel, intermittent hypoxia (IH) induces cell death. IH leads to HIF-1 activation responsible for ER stress (**A**), MAM disruption (**B**), Ca^2+^ homeostasis alteration, mitochondrial dysfunction (**C**), and cardiomyocyte death.

This study showed that, in human and rodent myocardial samples, IH induced a pro-apoptotic ER stress that was dependent on HIF-1. We further demonstrated that HIF-1 was involved in ER-mitochondria contacts disruption, ER-mitochondria Ca^2+^ fluxes alteration and mitochondrial function impairment. All together, we highlighted a new mechanistic role for HIF-1 in IH-induced cardiomyocytes apoptosis (Figure 7). 

### 4.1. HIF-1 Is Involved in IH-Induced ER Stress and Subsequent Cardiomyocyte Death

As previously demonstrated by a correlation between HIF-1α expression and the severity of SDB [9], we showed that HIF-1α expression had increased in atrial biopsies from patients with SDB (highest quartile) compared with individuals without SDB (lowest quartile). CHOP expression was also increased in this population [9], and we showed, for the first time, that the ER stress sensor, Grp78, was also increased in atrial biopsies from patients with SDB compared with non-SDB patients. Using our murine model of chronic IH, we demonstrated that HIF-1 plays a major role to trigger IH-induced ER stress and apoptosis, since IH-induced expression of both Grp78 and CHOP, as well as apoptosis, were prevented in HIF-1α^+/−^ mice (Figure 7A).

These results are in accordance with previous studies demonstrating, first, that chronic exposure to IH induces pro-apoptotic ER stress in various organs such as liver [42], brain [43], kidney [44], lung [45] and heart [25,41]; and second, that direct (tauroursodeoxycolic acid) or indirect (adiponectin, metallothionein) ER stress inhibition reduces IH-induced cardiomyocyte apoptosis [7,25,46]. HIF-1 activation is known to be one of the key mechanisms responsible for the deleterious effects of chronic IH [20], and previous studies have demonstrated a reciprocal deleterious link between HIF-1 activation and ER stress under IH in myocardium. Indeed, ER stress inhibition limited IH-dependent HIF-1 activation [25] and, conversely, IH failed to induce ER stress (i.e., CHOP expression) in HIF-1α^+/−^ mice [9], as well as in mice treated with curcumin, a natural inhibitor of HIF-1 [10]. This tight interaction between HIF-1 activation and ER stress has been described as a central mechanism involved in IH-induced cardiomyocyte apoptosis [10] and susceptibility to myocardial ischemia–reperfusion [25]. In the present work, we aimed to delve deeper into the mechanisms of cardiomyocyte death and focus on the interaction between ER and mitochondria.

### 4.2. HIF-1 Is Involved in IH-Induced MAM and Ca^2+^ Homeostasis Alterations

ER-mediated cardiomyocyte contractile integrity and homeostasis must be accompanied by mitochondrial ATP generation, which is achieved, at least in part, by propagation of the Ca^2+^ signal from ER to mitochondria through ER-MAM [47]. In this study, we demonstrated that IH induces a disruption of the most studied and representative physical interaction between ER and mitochondria (i.e., VDAC/IP3R1/Grp75) [48] (Figure 7B). We also observed an inability of the ER to release Ca^2+^ to mitochondria in response to histamine associated with an increase in cytosolic and ER Ca^2+^ overload. These IH-induced MAM and Ca^2+^ homeostasis disturbances may explain, in part, the deleterious effects of IH observed in the heart. Indeed, local Ca^2+^ transport from the ER to mitochondria is known to control other Ca^2+^ sensitive mechanisms, such as mitochondrial ATP production [49], ROS generation and systemic Ca^2+^ homeostasis [47]. Consequently, an alteration of MAM integrity is recognized to induce ER stress [50] and/or apoptosis [51]. Different proteins or stimuli have been described to impact MAM integrity allowing to directly or indirectly modulate their activity and cellular response to several stresses [26]. Very interestingly, the modulation of MAM integrity and its consequence is dependent on the stress. For example, Paillard et al. demonstrated that acute MAM disruption (by blocking Grp75) was protective against myocardial ischemia-reperfusion injury by avoiding Ca^2+^ mitochondrial overload, mPTP opening and apoptosis [33]. Conversely, the same team recently demonstrated that reduction in ER-mitochondria Ca^2+^ transfer by MAM disruption induces early mitochondrial dysfunction and contributes to chronic disease such as diabetic cardiomyopathy [34]. Indeed, it has been shown that VDAC-IP3R disruption is responsible for early mitochondrial bioenergetics dysfunctions and associated ER stress, contributing to the development of the pathology [34]. In the present study, we demonstrated that 3 weeks of IH induces HIF-1-dependent MAM alterations (Figure 7B). Indeed, IH induces MAM disruption and alteration of Ca^2+^ ER-mitochondria transfers in HIF-1α^+/+^ mice, with no effect in HIF-1α deficient mice. Moreover, when cardiomyoblasts were stimulated with CoCl_2_ (at a pro-apoptotic dose), HIF-1α was overexpressed and MAM integrity was also altered. To our knowledge, this is the first time that HIF-1 has been highlighted to interfere with MAM integrity. Interestingly, in both in vivo and in vitro models, HIF-1α expression is associated with a decrease in Grp75 that plays a primordial role in MAM integrity and especially, in VDAC and IP3R1 interaction. Taken together, these results suggest that HIF-1 activation could alter MAM through Grp75 expression modulation. Currently, only one study has evidenced a link between HIF-1 and Grp75. This study demonstrated that, upon oxygen limitation, HIF-1α and Grp75 interact at the outer mitochondrial membrane, indicating that the HIF-1α signaling pathway is not restricted to the nucleus [52]. Grp75 is mainly described as a key protein expressed at the MAM interface which regulates ER–mitochondrial Ca^2+^ [53]. Thus, we suggest here that, under chronic IH, HIF-1α interplays with Grp75, altering MAM integrity, leading to the alteration of Ca^2+^ homeostasis and ultimately cardiomyocyte death. This does not exclude that HIF-1, under chronic IH, could increase the expression of genes encoding for Grp75 modulators. However, further experiments need to be performed to better understand the specific and direct or indirect role for HIF-1 on Grp75 and the MAM complex.

### 4.3. HIF-1 Is Involved in IH-Induced Mitochondrial Respiration Alteration

IH induces a decrease in mitochondrial respiration that is prevented in HIF-1α^+/−^ mice, demonstrating a specific role for HIF-1 in IH-induced mitochondrial function alteration (Figure 7C). The impact of HIF-1 activation on mitochondrial respiration is consistent with its well-recognized role in the mechanism primarily involved in mitochondrial metabolic reprogramming under hypoxia [54,55]. Indeed, HIF-1 is described to support anaerobic ATP production through glycolysis activation and OXPHOS downregulation by several ways, such as replacing the respiratory chain complex, inhibiting entry into the tricarboxylic acid cycle, and utilizing glutamine-derived carbon [56]. Importantly, HIF-1, by altering MAM and disturbing both ER-mitochondria transfers and cytosolic and ER Ca^2+^ loading after ER leakage, could alter mitochondrial function, thereby promoting cardiomyocyte apoptosis. Indeed, as previously described, MAM plays an important role in Ca^2+^ hotspots management and mitochondrial integrity and function [57]. Preserving mitochondrial function is a key point for keeping the mPTP closed and avoiding apoptosis [58]. Here, we observed that HIF-1 is responsible for early mPTP opening, suggesting that IH-induced MAM alteration also triggers apoptosis by mPTP early opening. Moreover, the global Ca^2+^ homeostasis alteration generated by IH can alter mitochondrial function in different ways. Cytosolic overload has been previously associated with an increase in Na^+^/Ca^2+^ exchanger (NCX1) expression, which is encoded by HIF-1 [59] and known to predispose to ROS-mediated myocardial injury [60]. Finally, cytosolic Ca^2+^ overloading contributes to Serca2a activation by phospholamban phosphorylation that could explain Ca^2+^ rapid accumulation in ER and subsequent ER stress [61]. Otherwise, it has been demonstrated that HIF-1α overexpression promotes PLB phosphorylation and alters Ca^2+^ handling [62].

## 5. Conclusions and Perspectives

We demonstrated that chronic IH alters ER-mitochondria contacts, disturbs Ca^2+^ fluxes between the two organelles, decreases mitochondrial respiration and favors apoptosis. All these IH-induced perturbations occur in an HIF1-dependent manner and can lead to pro-apoptotic ER stress and cardiomyocyte death. These findings provide new evidence for the deleterious role of HIF-1 in IH-induced cardiomyocyte apoptosis, and strongly suggest that HIF-1 modulation may limit the cardiac deleterious effects of SDB. These results suggest that inhibiting ER stress, modulating mitochondrial respiration and/or function as well as energetic metabolism status could be an alternative or a complementary therapeutic strategy in SDB patients with a cardiovascular risk. In the preclinical model, we previously demonstrated the beneficial effect of direct (tauroursodeoxycholic acid) or indirect (exercise, curcumin) ER stress inhibitor [10,25,41]; however, others studies need to be performed to better characterize the impact of IH on energy metabolism and mitochondrial function and dynamics. HIF-1 is a transcriptional factor leading to adaptive and maladaptive responses depending on the hypoxic stimulus. Other researchers, and we, have clearly demonstrated that chronic HIF-1 activation by IH is deleterious. Thus, further studies need to specifically characterize HIF-1 activation (post-translational modification, localization, etc.) before suggesting its modulation in SDB patients.

## 6. Study limitations

Rhod-2, Fluo-4 and Fluo-5 are non-ratiometric probes. They were used to detect a modification in organelle Ca^2+^ content relative to a basal state. Even if they did not inform on organelle content, these probes have been previously demonstrated to detect Ca^2+^ transfers between organelles, when they were incubated two per two simultaneously. Rhod-2, which has previously been demonstrated to co-localize with mitochondria in cardiomyocytes [33], was incubated simultaneously with Fluo-4 or Fluo-5, before stimulating ER Ca^2+^ leakage by IP3R1. This allowed the measurement of Ca^2+^ variations in ER and cytosol at the same time, than in mitochondria for each sample. Ratiometric probes such as Fura-2, Indo-1 are used to measure calcium traffic in whole cells. However, in the context of calcium traffic in MAM, further studies need to consider ratiometric organelle-specific probes such as mitochondria-targeted calcium ratiometric probe (mt-Fura 2), genetically encoded ratiometric ER–calcium sensor.

In this study, we only used males. SDB is also associated with cardiovascular disorders in women. However, it seems that the mechanisms involved are not the same as the ones involved in men, and it also appears that women are not sensitive to the same characteristic (i.e., hypoxic burden, reoxygenation, duration) of the hypoxic pattern [63]. Moreover, it has recently been demonstrated that HIF-1 activity could be modulated by estrogen and other sex hormones [64]. Thus, the impact of sexual hormones has to be deeply studied under IH, and female should be included in further studies [65].

## Data Availability

Data is contained within the article and supplemental material.

## Supplementary Materials

The following supporting information can be downloaded at: https://www.mdpi.com/article/10.3390/antiox11081462/s1.

## Aknowledgments

We thank “Fondation de France”, “Agir pour les Maladies Chroniques Fondation”, “ANR”, “Else Kröner-Fresenius-Stiftung Foundation”, “DFG” and the “Medical Faculty at the University of Regensburg” for their financial support.
