# Supplementary material for: Intermittent Hypoxia-Induced Cardiomyocyte Death Is Mediated by HIF-1 Dependent MAM Disruption
Source: Antioxidants (Basel). 2022 Jul 27;11(8):1462. doi: 10.3390/antiox11081462 (PMC9405320; doi:10.3390/antiox11081462)
Supplement: Supplementary file 1 [file antioxidants-11-01462-s001.zip › antioxidants-1799091-supplementary.pdf]

Supplemental Figure S1. Mean data of main parameters: Age, Gender, Body-mass index, kind of cardiac surgery (CABG: Coronary Artery Bypass Grafting; VR: Valve Replacement), NT-Pro-BNP and Apnea Hypopnea Index caracterizing control and sleep disordered breathing (SDB) patients enrolled in the study as previously described in *Moulin S et al. Can J Cardiol, 2020*

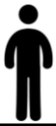

	Control	SDB	P value
Number	10	10	
Age (year)	68±3	73±2	
Gender (M/F)	10/0	10/0	
Body-mass index ($kg.m^{2}$)	28.4±0.8	29.9±1	
Cardiac surgery (CABG/VR/CABG-VR)	9/0/1	8/1/1	
NT-Pro-BNP (ng/ml)	1400±978	1000±372	
Polysomnographic data Apnea-hypopnea index (/h)	4.6±0.6	22±2.2	P<0.0001
